# Is the purinergic pathway involved in the pathology of COPD? Decreased lung CD39 expression at initial stages of COPD

**DOI:** 10.1186/s12931-018-0793-0

**Published:** 2018-05-28

**Authors:** Elisabet Aliagas, Mariana Muñoz-Esquerre, Ester Cuevas, Oriol Careta, Daniel Huertas, Marta López-Sánchez, Ignacio Escobar, Jordi Dorca, Salud Santos

**Affiliations:** 1 Pneumology Research Group, Institut d’Investigació Biomèdica de Bellvitge – IDIBELL, L’Hospitalet de Llobregat, Barcelona, Spain; 2 Department of Respiratory Medicine, Unit of Chronic Obstructive Pulmonary Disease, Bellvitge University Hospital, L’Hospitalet de Llobregat, Barcelona, Spain; 3 Department of Clinical Sciences, University of Barcelona, L’Hospitalet de Llobregat, Barcelona, Spain; 4 Department of Thoracic Surgery, Bellvitge University Hospital, L’Hospitalet de Llobregat, Barcelona, Spain; 5 Research Network in Respiratory Diseases (CIBERES), Madrid, Spain; 6 Department of Respiratory Medicine, Bellvitge University Hospital - IDIBELL, University of Barcelona, c/ Feixa Llarga s/n. CP 08907, L’Hospitalet de Llobregat, Barcelona, Spain

**Keywords:** COPD, CD39, ATP, Vascular remodeling, Inflammation

## Abstract

**Background:**

Extracellular adenosine triphosphate (ATP) is up-regulated in the airways of patients with chronic obstructive pulmonary disease (COPD), resulting in increased inflammation, bronchoconstriction, and cough. Although extracellular ATP levels are tightly controlled by nucleoside triphosphate diphosphohydrolase-1 (NTPDase1; also known as CD39) in the lungs, the role of CD39 in the pathology of COPD is unknown. We hypothesized that alterations in the expression and activity of CD39 could be part of the mechanisms for initiating and perpetuating the disease.

**Methods:**

We analyzed CD39 gene and protein expression as well as ATPase enzyme activity in lung tissue samples of patients with COPD (*n* = 17), non-obstructed smokers (NOS) (*n* = 16), and never smokers (NS) (*n* = 13). Morphometry studies were performed to analyze pulmonary vascular remodeling.

**Results:**

There was significantly decreased CD39 gene expression in the lungs of the COPD group (1.17 [0.85–1.81]) compared with the NOS group (1.88 [1.35–4.41]) and NS group (3.32 [1.23–5.39]) (*p* = 0.037). This attenuation correlated with higher systemic inflammation and intimal thickening of muscular pulmonary arteries in the COPD group. Lung CD39 protein levels were also lower in the COPD group (0.34 [0.22–0.92]) compared with the NOS group (0.67 [0.32–1.06]) and NS group (0.95 [0.4–1.1) (*p* = 0.133). Immunohistochemistry showed that CD39 was downregulated in lung parenchyma, epithelial bronchial cells, and the endothelial cells of pulmonary muscular arteries in the COPD group. ATPase activity in human pulmonary structures was reduced in the lungs of patients with COPD.

**Conclusion:**

An attenuation of CD39 expression and activity is presented in lung tissue of stable COPD patients, which could lead to pulmonary ATP accumulation, favoring the development of pulmonary inflammation and emphysema. This may be a mechanism underlying the development of COPD.

**Electronic supplementary material:**

The online version of this article (10.1186/s12931-018-0793-0) contains supplementary material, which is available to authorized users.

## Background

Chronic obstructive pulmonary disease (COPD) is characterized by small airway inflammation that causes airway obstruction, parenchymal destruction, and emphysema [1]. Cigarette smoking is the major risk factor for COPD, being responsible not only for local inflammation but also for the systemic inflammation that contributes to other comorbidities that affect disease severity and mortality [2]. Although most cases of COPD relate to smoking, not all smokers develop COPD, suggesting that the mechanisms initiating and perpetuating the disease are not fully understood [3]. Currently, no therapy effectively alters disease progression or improves survival due to the complexity of the signaling pathways that maintain the chronic inflammation and tissue destruction [4].

In recent years, numerous studies have generated data supporting the hypothesis that extracellular adenosine 5-triphosphate (ATP) is involved in the pathogenesis of COPD [5]. Specifically, increased pulmonary ATP levels have been found in mice with acute lung inflammation and emphysema following tobacco smoke exposure [6, 7]. Furthermore, red blood cells (RBC) represent an important physiologic compartment of ATP and the level of ATP in RBC in COPD patients is also significantly elevated [8]. In addition, in patients with COPD it has been shown that ATP accumulation stimulates the recruitment and activation of lung neutrophils and macrophages, which in turn enhance the release of proinflammatory and tissue-degrading mediators [9]. The release of ATP from neutrophils triggered by cigarette smoke activates the ATP receptor P2X_7_ for further activation of inflammasome pathways [10]. In animal models, it has been demonstrated that neutralizing intrapulmonary ATP levels or blocking airway specific ATP receptor subtypes can inhibit smoke-induced lung inflammation and confer protection from developing emphysema [7].

Extracellular ATP levels in mammalian tissues are tightly controlled by ecto-nucleotidases [11]. Among them, nucleoside triphosphate diphosphohydrolase-1 (NTPDase1; also known as CD39) is the most expressed purinergic ecto-enzyme in human alveolar and bronchial epithelia, submucosal glands, and fibroblasts [12]. CD39 is also found in the vascular endothelium, where it regulates homeostasis and is essential to angiogenesis and vasculogenesis [13]. Moreover, it regulates ATP-mediated P2 receptor signaling by hydrolyzing ATP/adenosine 5-diphosphate (ADP) to adenosine 5-monophosphate (AMP), which is then further metabolized by ecto-5′-nucleotidase/CD73 to adenosine [14]. Besides its general ecto-enzymatic functions, CD39 might modulate the function of neutrophil, macrophage, and dendritic cells by facilitating the hydrolisis of extracellular ATP and the further activation of the purinergic receptors [15, 16].

The role of CD39 in COPD has previously been investigated in an animal model of emphysema induced by tobacco exposure [17] and in inflammatory cells of COPD patients [17, 18], but its expression in human lung tissue is unknown. To better understand the mechanisms underlying COPD pathology we compared CD39 expression and activity in the lungs of patients with COPD (COPD group), non-obstructed smokers (NOS group), and never smokers (NS group). Our aim was to determine the expression and activity of CD39, the most efficient NTPDase for degrading ATP, in human lung samples between COPD, NOS, and NS groups.

## Methods

### Study subjects

We conducted a prospective study of 56 subjects who underwent lobectomy or pneumonectomy of a solitary pulmonary nodule at the Bellvitge University Hospital. Preoperative functional measurements were performed in all patients. Ten of them were excluded due to poor quality or insufficient sample. Therefore, 46 patients were included and according to their smoking history and the results of pulmonary function tests, subjects were classified as follows: (1) a COPD group, comprising 17 stable patients with COPD diagnosed according to the GOLD recommendations [1]; (2) an NOS group, comprising 16 smokers without COPD; and (3) an NS groups comprising 13 patients who had never smoked. Surgical lung samples were examined morphologically and histologically by immunolabeling experiments, molecular techniques, and enzymatic assays. All patients signed an informed consent form in accordance with the principles outlined in the Declaration of Helsinki, and the study was approved by the local ethics committee (CEIC, ref. PR330/15).

### Sample collection and processing

All lung tissue samples were obtained and processed immediately after surgery. Lung samples were divided into four and processed as follows: gene expression studies (frozen at − 80 °C in RNA Later; Qiagen, Hilden, Germany), morphological and immunological studies (immediately fixed, kept overnight in formalin, and embedded in paraffin), protein studies and enzymatic determination (directly frozen at − 80 °C), and in situ ATPase activity determination (fixed-formalin-lung samples were embedded in OCT freezing medium [Tissue Tek®; Sakura Finetek, Zoeterwoude, the Netherlands] and snap-frozen in dry ice). When sufficient lung samples could not be obtained for all studies, sample availability was prioritized in the given order.

### Quantitative real-time polymerase chain reaction analysis of CD39

Quantitative real-time polymerase chain reaction (qRT-PCR) was performed, as previously described [19]. The relative expression of CD39 in lung tissue samples was determined using *NTPD1* TaqMan Gene Expression Assays (catalog number Hs00969559_m1, Applied Biosystems) and RNA18S5 (Taqman Assay, Hs03928985_g1, Applied Biosystems) as the endogenous control for normalization. Data are expressed as a relative quantification (fold change ratio) of mRNA.

### Vascular morphometry

The histological and morphometric characteristics of pulmonary muscular arteries were analyzed in sections stained with hematoxylin and eosin, and orcein, using a computerized image analyzer, as previously reported [19, 20]. The areas occupied by the lumen, the intima, and the muscular layer are expressed as percentages of the total area encompassed by the external elastic lamina.

### Immunohistochemistry

Formalin-fixed, paraffin-embedded lungs were sectioned at a thickness of 4 μm. Sections underwent dewaxing, rehydration, antigen retrieval, and quenching of endogenous peroxidase activity. After three rinses in phosphate buffered saline (PBS), tissue sections were pre-incubated for 1 h at room temperature in 1% bovine serum albumin (Sigma-Aldrich, Sant Louis, Missouri, MO, USA), 0.2% gelatin (Merck, Darmstadt, Germany) and 0.1% triton (Sigma-Aldrich). Slices were then incubated overnight at 4 °C with mouse monoclonal antibody to human CD39 (Ref: ab178572, Abcam, Cambridge, UK) at 1/100, as the primary antibody. After three washes in PBS-triton, samples were incubated with the suitable avidin-biotin complex/peroxidase (Vectastain Elite ABC kit, Vector Laboratories, Burlingame, CA, USA), following the manufacturer’s protocol. Secondary antibody alone was routinely included to detect non-specific binding and placenta was used as positive control tissue for each experiment (data not shown). Nuclei were counterstained with haematoxylin, and the results were observed and photographed under a light Leica DMD 108 microscope (Leica Microsystems, Wetzlar, Germany). Evaluation and recording was by two investigators blinded to study conditions. Label intensity was scored as negative (−), weak (+), intermediate (++), or strongly positive (+++).

### Western blot

Frozen samples of lung tissue were homogenized and lysed in a RIPA lysis buffer (50 mM Tris-HCl pH 7.4, 150 mM NaCl, 1% Triton X-100, 0.5% sodium deoxycholate, 0.1% SDS, 1 mM EDTA) containing a protease inhibitor cocktail (Roche Molecular Systems, Pleasanton, CA). Protein from each sample (5 μg) was added to a 2× Laemmli sample buffer (Bio-Rad, Hercules, California, USA) containing 5% β-mercaptoethanol, which was boiled for 10 min at 56 °C, separated by 4–20% sodium dodecyl sulfate polyacrylamide gel electrophoresis, and electrophoretically transferred onto nitrocellulose membranes (Bio-Rad). Membranes were then blocked with 4% bovine serum albumin for 1 h at room temperature, washed with Tris-buffered saline-Tween 20, and incubated with mouse monoclonal (IMG17B5F11) anti-CD39 (1/1000; Abcam, cat:178572), or mouse monoclonal anti β-actin (1/4000; Abcam, cat: 8226) antibodies for 1 h at room temperature. Membranes were then washed, incubated with horseradish peroxidase-conjugated anti-mouse secondary antibodies (Dako, Carpinteria, CA, USA) for 1 h at room temperature, and developed using Enhanced ECL (Bio-Rad). β-actin was used for normalization of total protein in each lane.

Densitometry was performed and represented as a ratio of the pixel intensity of the CD39 illuminated protein compared with β-actin protein, using a multi-gage. Normalized values are expressed as the fold change form values obtained in control samples.

### In situ ATPase activity

Localization of ATPase activity was determined using the Wachstein/Meisel lead phosphate method [21]. Briefly, OCT tissue sections were rinsed in PBS and then pre-incubated for 1 h at room temperature in 50 mM Tris-maleate buffer, at pH 7.4, which contained 2 mM CaCl_2_ and 0.25 M sucrose. Enzymatic reaction was performed for 1 h at 37 °C in the same buffer, supplemented with 5 mM MnCl_2_, 2 mM Pb(NO_3_)_2_, 3% dextran T250, and 2.5 mM levamisole, to inhibit alkaline phosphatase activity and in the presence of 1 mM ATP as the substrate. For CD39 inhibition, 1 mM of NF279 (Tocris Bioscience, Bristol, UK) was added to both the pre-incubation and enzymatic reaction buffers. Substrate was omitted in control experiments and the reaction was revealed by incubation with 1% (NH_4_)_2_S *v*/v for exactly 1 min. Nuclei were counterstained with haematoxylin, before samples were dehydrated, mounted with DPX mounting medium, and observed and photographed under light microscopy.

### ATPase activity assays in tissue homogenates

Frozen samples of lung tissue were homogenized in a phosphate-free buffer containing 20 mM HEPES (4-(2-hydroxyethyl)-1-piperazineethanesulfonic acid) at pH 7.4 with 250 mM sucrose, 1 mM EGTA, 1 mM MgCl, 0.3 mM PMSF, and 1 mM DTT. ATPase activity was determined using a malachite green phosphate assay kit (BioAssay Systems, Hayward, California, CA, USA). Lung tissue homogenates (5 μg) were added to an incubation mixture containing 160 mM Tris-HCl (pH 7.5), 10 mM CaCl_2_, and 5 mM levamisole, as alkaline phosphatase inhibitor. The reaction started by adding 1 mM ATP as substrate for 1 h at 37 °C. Controls to determine non-enzymatic inorganic phospathe (Pi) accumulation were performed by incubating the samples either with or without the substrate. All samples were analyzed in triplicate, and the specific activity is expressed as arbitrary units (AU).

Further details on methods are reported in the Additional file 1, available at Respiratory Research online.

### Statistical analysis

Continuous variables were compared by Student’s t-test and are expressed as mean ± standard deviation (SD) or by Mann–Whitney *U*-test and expressed as median and interquartile range, regardless of whether a normal distribution was assumed or not, respectively, based on the Kolmogorov–Smirnov test. Qualitative variables were compared with the chi-square. Comparisons between groups were evaluated by one-way analysis of variance (ANOVA) or Kruskal-Wallis test, as appropriate, and an overall *p*-value was calculated. Adjusted analyses were performed using unbalanced demographic variables as covariate (gender, tobacco exposure and the presence of diabetes mellitus, *p* < 0.05). Spearman’s correlation was also used to assess the relationship between biological variables (the percentage of intimal area in pulmonary arteries and the CD39-related gene expression). Statistical analysis was performed using IBM SPSS, version 19.0 (IBM Corp., Armonk, NY, USA). A *p*-value < 0.05 was considered statistically significant.

### Study endpoints and power calculation

The primary study endpoint was the difference in CD39 gene expression in the COPD group compared to the NS group. Assuming a standard deviation of 0.77 × 10^− 3^ in the CD39 mRNA (ddDt) of the control group, a sample size of 13 subjects per group was needed to detect a minimal difference of 0.70 × 10^− 3^ between groups with an 80% power and a two-tailed *p*-value less than 0.05. Allowing for an approximate 15% dropout rate (e.g. inadequate samples for measurements), including 15 subjects per group was expected to be sufficient to provide data from 13 samples for each analysis. Secondary endpoints included between-group comparisons of CD39 protein expression and immunoreactivity in the lungs.

## Results

Lung samples from 56 patients undergoing lung resection were included, of which 10 samples were discarded due to poor quality or insufficient samples. Therefore, 46 patients were included (17, 16, and 13 in the COPD, NOS, and NS groups, respectively). There were no significant differences in baseline characteristics between groups, except for gender, tobacco exposure, and the presence of diabetes (Table 1), so these were included as covariates in all adjusted analyses.

**Table 1:** Baseline characteristics and pulmonary function parameters by study group

Characteristic	COPD (*N* = 17)	NOS (*N* = 16)	NS (*N* = 13)	*p*-value
Male gender, n (%)	15 (88)	16 (100)	5 (38)	< 0.001
Age, years	64.4 ± 7	61.0 ± 11	61 ± 14	0.617
BMI, kg/m^2^	25.4 ± 4.2	27.4 ± 4.5	27.2 ± 4.2	0.364
Current smokers, n (%)	3 (17)	9 (56)	–	< 0.001
Pack-years	51.47 ± 15.52	39.87 ± 19.09	–	0.072
Diabetes Mellitus, n (%)	1 (6)	6 (37.5)	–	0.005
Fast basal glucose, mmol/L	5.2 [4.7–6.6]	6.5 [5.2–7.3]	5.3 [4.9–6.3]	0.056
Systemic hypertension, n (%)	8 (34.8)	7 (43.7)	5 (31.3)	0.896
Inhaled CS, n (%)	5 (29.0)	0 (0)	1 (7)	0.039
FVC, L	3.3 ± 0.7	4 ± 0.9	3.5 ± 1	0.079
FVC, % predicted	89 ± 14	101 ± 17	111 ± 23	0.008
FEV_1_, L	1.9 ± 0.5	3 ± 0.6	2.7 ± 0.8	< 0.001
FEV_1_, % predicted	64 ± 16	97 ± 14	106 ± 23	< 0.001
% FEV_1_/FVC	57 ± 10	76 ± 5	77 ± 5	< 0.001
DL_CO_,% predicted	69 ± 14	89 ± 17	93 ± 18	< 0.001
Leukocytes count, x10E9/L	8.7 ± 1.9	8.2 ± 2	6.9 ± 1.5	0.038
C-reactive protein, mg/L	5.4 [2.1–10.6]	2.1 [1.0–10.9]	1.8 [1–2.7]	0.028

Data are presented as mean ± SD or median [25th–75th percentile]

*COPD*Chronic Obstructive Pulmonary Disease, *NOS* Non-obstructed smokers, *NS* Never smokers, *BMI* Body mass index, *CS* Corticosteroids, *FVC* Forced vital capacity, *FEV*_*1*_ Forced expiratory volume in one second, *DL*_*CO*_ Diffusing lung capacity for carbon monoxide. A *p*-value < 0.05 was considered statistically significant

### Gene expression in lung tissue samples

**Fig. 1:**
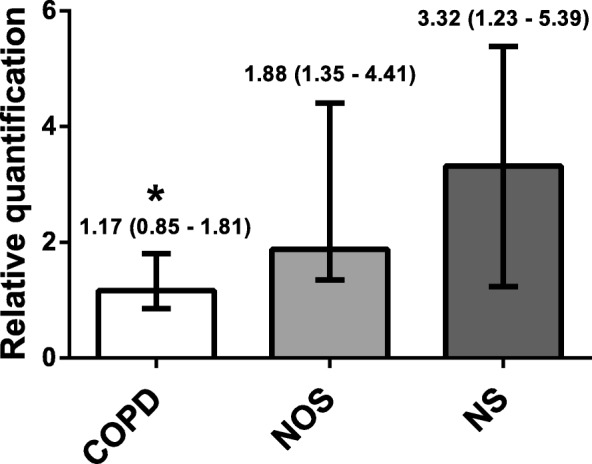
NTPDase1/CD39 Gene Expression in Human Lung Tissue Samples. Relative mRNA levels of CD39 analyzed in the COPD (*n* = 15), NOS (*n* = 13) and NS (*n* = 11) groups, normalized to 18 S mRNA levels and expressed as fold changes. Data are expressed as median ± rank. *Significantly different from NS and NOS (*P* < 0.05). Abbreviations: NTPDase1, nucleoside triphosphate diphosphohydrolase-1; COPD, Chronic obstructive pulmonary disease; NOS, Non-obstructed smokers; NS, Never smoker

The qRT-PCR analyses showed that CD39 was significantly downregulated in the COPD group compared with both the NOS and NS groups (1.17 [0.85–1.81] vs 1.88 [1.35–4.41] and 3.32 [1.23–5.39], respectively, *p* = 0.037) (Fig. 1). In addition, there was a statistically significant inverse correlation between CD39 gene expression in lung tissue and serum C-reactive protein levels (Spearman’s rho = − 0.462, *p* = 0.005).

### Morphometric measurements of intimal thickening

The results of morphometric measurements performed in pulmonary muscular arteries are shown in Table 2. As shown, the intimal layer was enlarged in the COPD group compared with the NOS and NS groups, in agreement with previous studies [19, 20]. However, the thickness of the muscular layer in these arteries did not show any significant between-group differences. There was a significant correlation between the intimal thickening of pulmonary muscular arteries (expressed as percentage of total area) and lung CD39 gene expression (Spearman’s rho = 0.39, *p* = 0.032).

**Table 2:** Morphometric characteristics in pulmonary muscular arteries by study group

	COPD (*N* = 12)	NOS (*N* = 8)	NS (*N* = 12)	*p*-value
N° of arteries measured by patient	10 ± 4	12 ± 6	8 ± 6	0.203
Artery diameter, μm	306 ± 65	349.33 ± 57.7	333.51 ± 18.8	0.186
Lumen area, % total area	27.7 ± 7.1	35.1 ± 8.2	36.2 ± 9.8	0.046
Intimal area, % total area	37.3 ± 7	29.4 ± 7.1	26.6 ± 11.5	0.018
Muscular area, % total area	35 ± 4.2	35.5 ± 5.9	37.2 ± 11.2	0.782
Index of narrowing^a^	0.299 ± 0.032	0.301 ± 0.024	0.307 ± 0.269	0.831

*COPD* Chronic Obstructive Pulmonary Disease, *NOS* Non-obstructed smokers, *NS* Never smokers

Data are presented as median ± standard deviation. *P*-values are for the overall comparison by ANOVA

^a^Index of narrowing was estimated as the ration between the measured total area and the area extrapolated from the theoretical distended diameter: (theoretical diameter = length of the external elastic lamina/pi (π))

### Immunolabeling

**Fig. 2:**
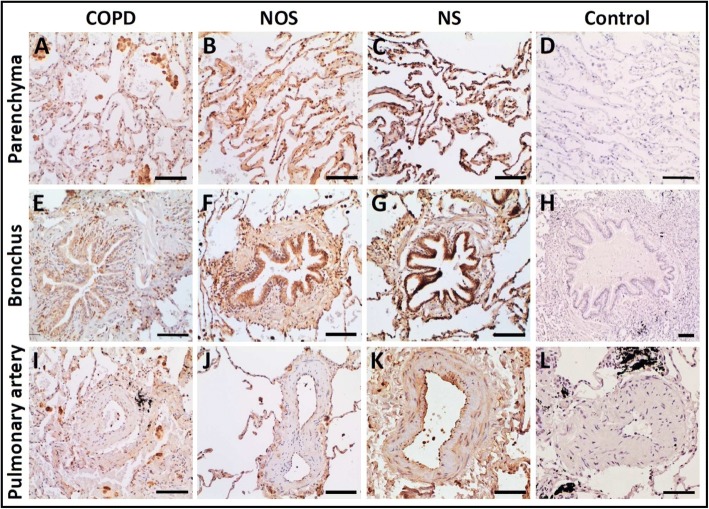
Immunolocalization of NTPDase1/CD39 in Human Lung Structures: Parenchyma (**a**–**d**), Bronchi (**e**–**h**), and Pulmonary Arteries (**i**–**l**). CD39 was downregulated in lung parenchyma, bronchial epithelial cells, and endothelial cells of pulmonary arteries in the COPD group. Images are of representative histological slides from the COPD (*n* = 15), NOS (*n* = 13), and NS (*n* = 12) groups. The primary antibody was omitted in the control experiments in images D, H, and L. Scale bars = 100 μm. Abbreviations: NTPDase1, nucleoside triphosphate diphosphohydrolase-1; COPD, Chronic obstructive pulmonary disease; NOS, Non-obstructed smokers; NS, Never smoker

As expected, CD39 was expressed in lung parenchyma, bronchial epithelia, and the endothelial cells of muscular pulmonary arteries. In all these structures, CD39 immunolabeling was weaker in the COPD group than in the NOS and NS groups (Table 3). Representative images showing the downregulation of CD39 in the COPD group compared with the NOS and NS groups are shown in Fig. 2.

**Table 3:** Analysis of CD39 immunostaining intensity by study group

CD39 Stain Score for lung parenchyma	COPD	NOS	NS
+++	1 (6,66)	0 (0)	1 (8,33)
++	3 (20)	8 (61,53)	6 (50)
+	10 (66,66)	4 (30,78)	4 (33,33)
0	1 (6,66)	1 (7,69)	1 (8,33)
Total of Samples	15	13	12
CD39 Stain Score for the Bronchi	COPD	NOS	NS
+++	5 (12,82)	16 (25,4)	25 (40,32)
++	9 (23,08)	30 (47,62)	29 (46,77)
+	21 (53,84)	17 (26,98)	8 (12,9)
0	4 (10,25)	0 (0)	0 (0)
Total of Bronchus	39	63	62
Total of Samples	12	12	12
CD39 Stain Score for the Muscular Pulmonary Artery	COPD	NOS	NS
+++	20 (9,13)	7 (4,46)	19 (17,59)
++	38 (17,35)	52 (33,12)	39 (36,11)
+	80 (36,53)	58 (36,94)	43 (39,81)
0	81 (36,98)	40 (25,48)	7 (6,48)
Total of Pulmonary Artery	219	157	108
Total of Samples	15	13	12

*COPD* Chronic Obstructive Pulmonary Disease, *NOS* Non-obstructed smokers, *NS* Never smokers. Number of samples, Stain Intensity and Percentage are shown for each result

### Protein expression analysis by western blot

**Fig. 3:**
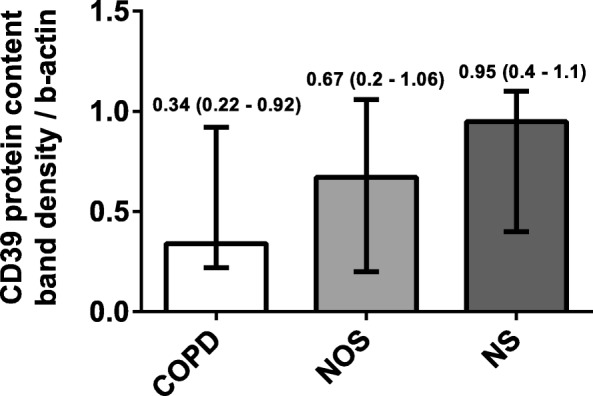
Lung NTPDase1/CD39 Protein Expression Divided by Groups. Analyses of total lung tissue showing the band densities of CD39 in the COPD (*n* = 13), NOS (*n* = 12), and NS (*n* = 12) groups. CD39 protein levels were lower in the COPD group. Data are expressed as median ± rank. Abbreviations: NTPDase1, nucleoside triphosphate diphosphohydrolase-1; COPD, Chronic obstructive pulmonary disease; NOS, Non-obstructed smokers; NS, Never smoker

Total CD39 lung content was measured by Western blot. Densitometric analyses of the bands showed that CD39 band density was lower in the COPD group (0.34 [0.22–0.92]) compared with the NOS group (0.67 [0.32–1.06]) and the NS group (0.95 [0.4–1.1]) (Fig. 3; *p* = 0.133). However, the differences were not statistically significant.

### In situ enzyme histochemistry and ATPase activity in lung tissue homogenates

**Fig. 4:**
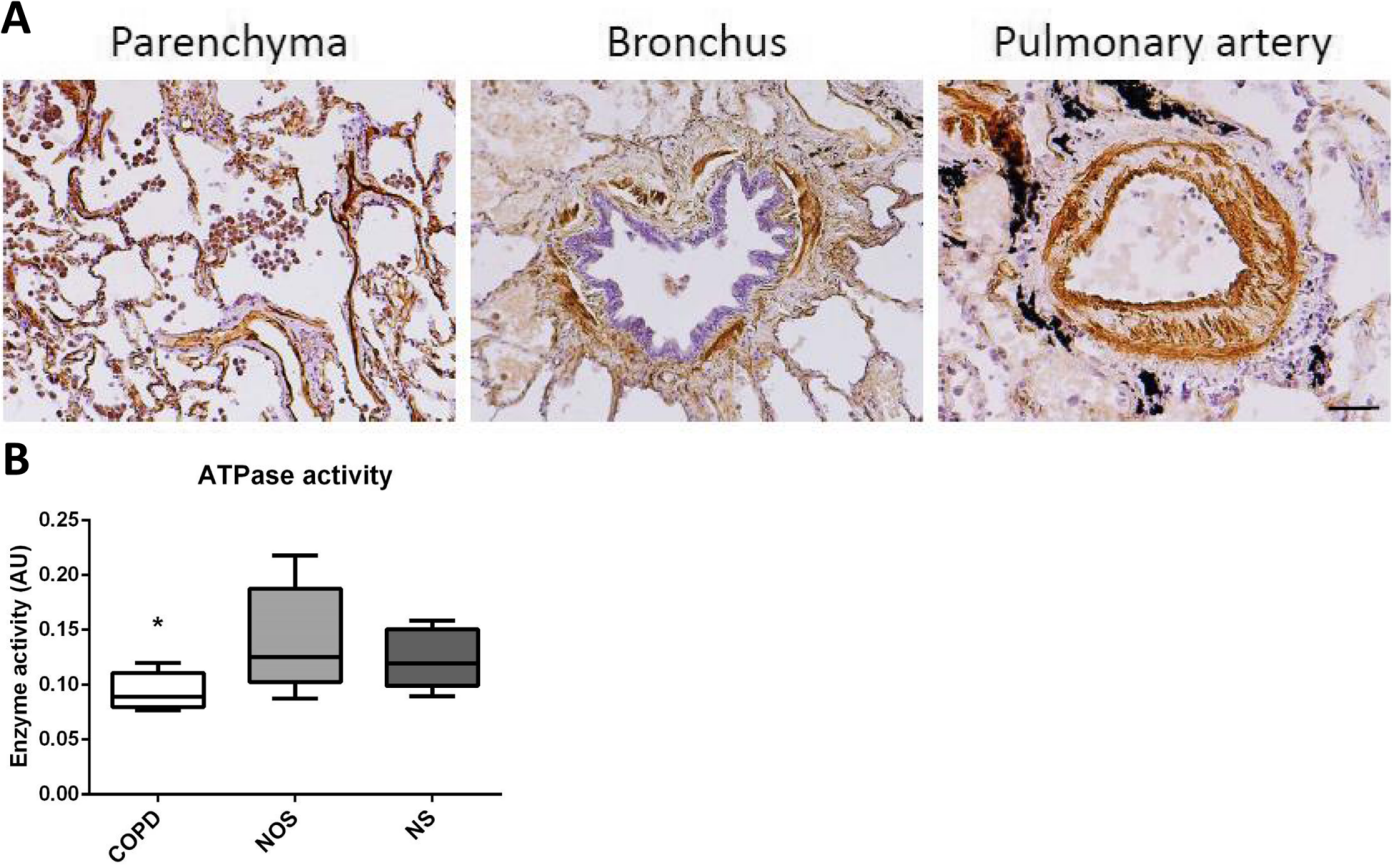
In Situ Enzyme Histochemistry and ATPase Activity in Lung Tissue Homogenates. **a** Examples of enzyme in situ histochemistry in lung structures such as parenchyma, bronchus and pulmonary artery from NOS. The localization of ATPase activity is showed as dark brown deposits. Scale bar = 100 μm. **b** ATPase enzyme activity in tissue homogenates for the COPD, NOS, and NS groups (*n* = 7, per group). Experiments were performed in triplicate for each sample. Data are represented in arbitrary units (AU). * Significant differences at *p* < 0.05. Abbreviations: COPD, Chronic obstructive pulmonary disease; NOS, Non-obstructed smokers; NS, Never smoker

To determine if the differences in CD39 gene and protein expression produced changes in enzyme activity between groups, we assessed ATPase activity. Our results confirmed that ATPase activity was present in the lung parenchyma, bronchi, and pulmonary arteries (Fig. 4a). The specific ATPase inhibitor, NF279, also effectively abolished those activities. Finally, quantification of ATPase activity in lung homogenates demonstrated that patients with COPD had decreased ATP hydrolysis (Fig. 4b; *p <* 0*.*01)).

## Discussion

Increased extracellular levels of ATP in the pulmonary samples of patients with COPD have been linked to inflammation and emphysema [22, 23]. Few studies have addressed the expression or activity of pulmonary NTPDases as CD39 in COPD. The focus of our study was to determine the expression and activity of CD39, the most efficient NTPDase for degrading ATP, for the first time in human lung tissue between COPD, NOS, and NS groups. Although the patients studied have established COPD, the stage of their disease is not advanced but mild-moderate, and at this time molecular changes may be more representative of the initial pathogenic injuries.

Our main result was that CD39 gene expression decreased in the COPD group. Data regarding CD39 protein levels also reflect this trend, but it is not statistically significant. Consistent with our observations, Kratzer et al. [24] reported a significant downregulation of CD39 expression in the lung tissue of rats exposed to cigarette smoke. By contrast, Lazar et al. [17] observed increased CD39 expression in the leukocytes of a COPD group compared with smoker and NS groups. These seemingly contradictory findings might be explained by the difference in the compartmentalization of CD39 expression in COPD (lung tissue versus inflammatory cells) [17]. The increased CD39 expression in leukocytes could compensate for the attenuated CD39 lung expression and combat airway inflammation in COPD. Tan D et al., [18] demonstrated enhanced expression of CD39 on circulating T-cell subsets in patients with an exacerbation of COPD compared with patients with stable COPD. Our results are not at odds with those of these two previous studies; in fact they complement our current knowledge of CD39 in COPD. We show that the expression of CD39 in lung tissue in patients with COPD (in the initial phase) is diminished; therefore, as found in the animal model of Lazar et al. [17], administering a CD39 analogue might protect against the development of pulmonary emphysema and inflammation in human patients as well. Our study did not analyze the expression of CD39 in the pulmonary or systemic inflammatory cells. However, the increase in CD39 expression in airway and systemic inflammatory cells suggested by both articles may be the compensatory mechanism for acute exposure to tobacco [17] or infection [18].

Attenuation of lung CD39 expression in COPD could be responsible for the increased ATP levels detected in pulmonary samples from patients with COPD [23]. Interestingly, it has been reported that the high amounts of ATP detected during asthma exacerbations could be due to reduced leukocyte expression of ectonucleotide pyrophosphatase/ phosphodiesterase 1 (ENPP1), another ATP degrading enzyme [25]. Furthermore, CD39 has been shown to be involved in the pathogenesis of allergic inflammation by modulating dendritic cell function [26].

We compared the localization and expression of CD39 in the lung samples of COPD, NOS, and NS groups. In the lung, CD39 is mainly localized in the parenchyma, bronchial epithelial cells, and endothelial cells. In patients with cystic fibrosis, CD39 expression has been detected in alveolar and bronchial epithelial cells and in endothelial cells [12], and it has also been reported to be expressed in smooth muscle cells, platelets, and immune cells [27]. Our results show that lung CD39 expression, was diminished in all lung structures of patients with COPD. This pulmonary scenario, with a high ATP niche, favors proinflammatory responses of the innate immune system via the activation of ATP receptors (e.g., P2X) expressed in human airways [28] and on the surface of immune cells [29].

Enzymatic ATPase activity was demonstrated in lung tissue samples. Previously, pulmonary ATPase activity has been shown in the bronchoalveolar lavage fluid of humans and mice exposed to cigarette smoke [17]. As expected, ATPase activity was found in the same structures where it was immunolocalized (i.e., the parenchyma, bronchi, and pulmonary artery). In addition, when we quantified ATPase activity and compared it between groups, activity was significantly lower in the COPD group. Decreased CD39 activity has been shown in tissue compartments due to oxidative stress and proinflammatory cytokines like tumor necrosis factor-α [30]. Recently, it has been suggested that pharmacological therapies aimed at neutralizing ATP or blocking its receptors could be used to treat COPD. To date, the efficacy of this therapy has only been demonstrated in mice [7].

We also showed that downregulation of CD39 gene expression correlated with increased blood C-reactive protein levels. Systemic inflammation, which has previously been described in a high proportion of patients with stable COPD, has also been associated with a worse prognosis and higher mortality [31]. In the present research, we showed an inverse association between both biomarkers. This finding provides further support for the compensatory theory of the increase in CD39 in circulating blood inflammatory cells associated with the exacerbation of COPD described in a previous article [18].

We also observed that lung samples with fewer CD39 gene expressions have greater vascular intimal thickening. We hypothesized that downregulation of pulmonary CD39 led to ATP accumulation and thereby created a beneficial environment for inflammation and remodeling. Recently, attenuated CD39 enzyme activity in endothelial cells has been associated with vascular remodeling in patients with idiopathic pulmonary arterial hypertension [32]. To further investigate the contribution of decreased CD39 levels to the pathobiology of pulmonary hypertension in COPD, it would be interesting to study CD39 expression in muscular pulmonary arteries and its relationship to vascular remodeling in patients with pulmonary hypertension secondary to COPD.

Several limitations of this study need to be discussed. First, there were few females in both the COPD and NOS groups (most patients with major smoking habits were male). This makes it difficult to draw conclusions about the role of sex. Second, the study population included those with primary, localized, and treatable lung cancer; therefore, lung cancer could be a possible introduced bias. However, we assumed that any bias introduced by the presence of lung carcinoma would be the same across all subjects, and this must be considered when comparing CD39 expression and differences between the COPD, NOS, and NS groups. On the other hand, this is the only model that allows us to correlate clinical and analytical data and data on lung function, in stable patients and in those at the initial stages of the disease, with the structural and biological characteristics of the lung tissue from the same patients.

Finally, due to the study’s observational design, no causal conclusions can be drawn, and we may only state that there was an association between the presence of COPD and the low expression of CD39.

## Conclusions

Our data show an attenuation of CD39 expression and activity in lung tissue of stable mild COPD patients. These results could explain the pulmonary accumulation of extracellular ATP in the lung, previously demonstrated in COPD patients, which confers increased proinflammatory responses favouring the development of emphysema. Further studies are needed to better understand the real role of CD39 in this COPD context.

## Additional file

Additional file 1: Supplementary data. (PDF 360 kb)

## Abbreviations

ANOVA: Analysis of variance
ATP: Adenosine 5-triphosphate
AU: Arbitrary units
COPD: Chronic obstructive pulmonary disease
DTT: 1,4-Dithiothreitol
EGTA: Ethylene glycol-bis(β-aminoethyl ether)-N,N,N′,N′-tetraacetic acid
NOS: Non-obstructed smokers
NS: Never smoker
NTPDase: Nucleoside triphosphate diphosphohydrolase
PBS: Phosphate buffered saline
PMSF: Phenylmethanesulfonyl Fluoride
SD: Standard deviation
