# Supplementary material for: Is the purinergic pathway involved in the pathology of COPD? Decreased lung CD39 expression at initial stages of COPD
Source: Respir Res. 2018 May 28;19:103. doi: 10.1186/s12931-018-0793-0 (PMC5972409; doi:10.1186/s12931-018-0793-0)
Supplement: Supplementary file 1 — Supplementary data. (PDF 360 kb) [file 12931_2018_793_MOESM1_ESM.pdf]

RERE-D-18-00011

Is the purinergic pathway involved in the pathogenesis of COPD? CD39: a new possible therapeutic target of COPD.

Supplementary Methods

Study Subjects

Patients who were scheduled for pulmonary nodule surgery were selected for the study through an exhaustive review of their clinical history. The following were excluded: those who presented severe systemic comorbidities, atelectasis or obstructive pneumonitis, those who had received chemotherapy or radiotherapy prior to surgery, those with diagnoses of chronic pulmonary disease other than COPD or those who did not sign the consent to participate in the study. Patients included were classified into the three study groups based on their pulmonary function tests prior to the intervention and on their tobacco consumption.

Sample Collection and Processing

Samples were collected and processed in accordance with the protocols published in the CIBERES biobank (biobancopulmonar.ciberes.org/es/protocolos).

RNA isolation and reverse transcription

Total RNA was isolated using TRIzol reagent (Life Technologies, Paisley, UK). Genomic DNA digestion and RNA purification was performed with the DNasa I amplification grade kit (Life Technologies). RNA purity was tested measuring the A260/A280 ratio by spectrophotometer (NanoDrop Microvolume UV-Vis Spectrophotometer with Wi-Fi, Thermo Fisher Scientific, Waltham, Massachusetts, MA, USA) on all the samples prior to perform qRT-PCR. RNA used had to have the A260/A280 ratio between 1.8 and 2.1. Total purified RNA (1 µg) was reversely transcribed into complementary DNA (cDNA) using the High capacity cDNA kit with RNAse inhibitor according to the manufacturer's instructions (Applied Biosystems, Foster City, CA, USA). Reactions were incubated in a PCR Thermocycler (MyCycler^{TM} Thermal Cycler System With Gradient Option, BIO-RAD,

Hercules, California, USA) at 25 ˚C for 10 minutes, 37 ˚C for 120 minutes and 85 ˚C for 5 minutes.

Quantitative RT-PCR Analysis of CD39

Quantitative real-time-PCR (qRT-PCR) was performed to determine the gene expression of *NTPD1* in lung tissue samples. TaqMan quantitative RT-PCR assays for *NTPD1* was performed in triplicate on cDNA samples in 384-well optical plates using an ABI Prism 7900HT Real Time PCR System (Applied Biosystems, Life Technologies, Waltham, MA). For each 10-µL TaqMan reaction, 1 µL of cDNA was mixed with 3.5 µl of RNAse free water, 0.5 µL 20X TaqMan Gene Expression Assays (catalogue number Hs00969559_m1) and 5 µL of 2X TaqMan Universal PCR Master Mix (Applied Biosystems). Parallel assays for each sample were carried out using probes for RNA18S5 (Taqman Assay, Hs03928985_g1, Applied Biosystems). A common calibrator for each plate was used. The reactions were carried out using the following parameters: 50 °C for 2 minutes, 95 °C for 10 minutes, and 40 cycles of 95 °C for 15 seconds and 60 °C for 1 minute. Finally, all TaqMan PCR data were captured using the SDS software v2.4 (Applied Biosystems) and analyzed with the double-delta cycle threshold (ΔΔCT) method using Expression Suite v1.0.3 software (Applied Biosystems). The ΔCT values represent normalized target gene levels with respect to the internal control. The ΔΔCT values were calculated as the ΔCT of each test sample minus the mean ΔCT of the calibrator samples. The fold change was determined using the equation 2(−ΔΔCT).

Immunohistochemistry Microscopy

Immunohistochemistry experiments were carried out in order to study the expression and the exact localization of CD39 in the lung. Briefly, formalin-fixed, paraffin-embedded lungs were sectioned. Sections of 4 µm were deparaffinised by xylol baths (3 x 10 minutes), rehydrated in decreasing concentrations ethanol series (3 x 100%, 3 x 96%, 70%) and rinsed in phosphate buffer saline (PBS). For antigen retrieval, slides were boiled in citrate buffer, pH 6.0 for 2 minutes using a pressure cooker. After 3 rinses in PBS, tissues were incubated in PBS containing 30% methanol and 1% hydrogen peroxide during 30 minutes for endogenous peroxidase quenching. After 3

rinses in PBS, sections were pre-incubated for 1 hours at room temperature in 1% bovine serum albumin (Sigma-Aldrich, Sant Louis, Missouri, MO, USA) 0.2% gelatin (Merck, Darmstadt, Germany) and 0.1% triton® X-100 (Sigma-Aldrich, Sant Louis, Missouri, MO, USA). Slices were then incubated overnight at 4°C with mouse monoclonal antibody to human CD39 (Ref: ab178572, Abcam, Cambridge, UK) at 1/100, as primary antibody. After three washes in PBS-triton, samples were incubated with the suitable avidin-biotin complex/peroxidase (Vectastain Elite ABC kit, Vector Laboratories, Burlingame, CA, USA), following the manufacturer's protocol. For immunohistochemistry experiments, we used a positive control for CD39 in placental tissue (data not shown) because it has been reported in the Protein Atlas that placental tissue has a high CD39 protein expression. Secondary antibody alone was routinely included to detect non-specific binding. Nuclei were counterstained with haematoxylin, and the results were observed and photographed under a light Leica DMD 108 light microscope (Leica Microsystems, Wetzlar, Germany. Evaluation and recording was by two investigators blinded to study conditions. Label intensity was scored as negative (-), weak (+), intermediate (++), or strong positive (+++).
